# Temperature and Composition Dependent Structural Evolution: Thermodynamics of Cu_n_Ag_135−n_ (n = 0–135) Nanoalloys during Cooling

**DOI:** 10.3390/molecules26206242

**Published:** 2021-10-15

**Authors:** Jinhan Liu, Naipeng Sun, Lin Zhang

**Affiliations:** 1 Key Laboratory for Anisotropy and Texture of Materials, Ministry of Education, Northeastern University, Shenyang 110819, China; 1910214@stu.neu.edu.cn; 2 Department of Materials Physics and Chemistry, School of Materials Science and Engineering, Northeastern University, Shenyang 110819, China; 3 The State Key Laboratory of Rolling and Automation, Northeastern University, Shenyang 110819, China; 1900603@stu.neu.edu.cn

**Keywords:** nanoalloy, core-shell, molecular dynamic, atomic packing

## Abstract

Molecular dynamics simulations are performed to investigate the changes of packing structures, and thermodynamic quantities including internal energy, entropy, and free energy are used to determine temperature regime and transition time of atomic packing structures. The simulation results show different packing structures as the component composition changes, and there are different packing patterns during cooling. For these Cu-Ag alloy clusters containing only a small number of atoms of Cu, they present FCC packing structures in different parts at high temperatures, and then there are transformations to icosahedral structures. With the increase in content of Cu atoms, there is a transition mechanism from molten state to icosahedron. When the content of Cu atoms is appropriate, core-shell structures can be formed at room temperature.

## 1. Introduction

During recent decades, bimetallic nanoclusters are of great significance in basic science and technological applications, such as magnetism, catalysis, and optics, and have attracted the attention of researchers [1,2,3,4,5,6,7]. The unusual physicochemical, electronic, and magnetic properties are mainly from differences in the atomic number of the group elements, elemental segregation effects, etc., and make those bimetallic clusters suitable for heterogeneous catalysis, sensors, and optoelectronic devices [8]. Therefore, studying of bimetallic nanoclusters is important for understanding and controlling the alloying at the nanoscale. Bimetallic nanoalloying particles can form a variety of isomers by combining atoms into different arrangements, and the mixture of different components also results in different characteristics [9]. It is found that many bimetallic nanoclusters can form core-shell structures [10]. When either one metal has a lower surface energy than the other or there is an atomic radius mismatch, the smaller atoms tend to occupy the cluster core to release the strain, whereas the larger atoms will move to the shell [11,12,13]. Compared with the traditional binary nanoalloys, on one hand, the core-shell structure of bimetallic nanoparticles can be modified by surface processing, i.e., depositing specific substances on the surface of the in-core particles, and, thus, giving the particles new functions. On the other hand, by the interactive modulation of the electronic structure between the core and shell atoms, it can form new surface structures and electronic structures [14,15]. Therefore, in the past few decades, nanoalloy particles with core-shell structure have attracted great interest and attention [16].

Silver nanoparticles present many distinct properties, such as high electrical conductivity, excellent photoelectricity, and antioxidant and antibacterial activity [17,18,19,20,21,22,23]. However, the high cost of silver nanoparticles makes it very limited in industrial production. As a typically eutectic alloy, when some of the Ag atoms in the nanoparticles are replaced with Cu atoms, alloying nanoparticles having the core-shell structures can be formed. It has been proved by experiment that a silver nucleus with a small size gradually agglomerated and can finally formed a core@shell structure of Cu@Ag with an increase in the concentration of metal sources [24]. The Ag-Cu nanoalloys are less expensive and have better electrical, optical, and catalytic properties [25]. Accounting for the fact that Cu nanoparticles have poor oxidation resistance, Ag atoms are wrapped on the surface of the Cu nuclei to prevent Cu oxidation, which also constitutes the commonly known Cu-Ag nanoclusters with core-shell structure, labeled as Cu@Ag nanoclusters. Liu et al. found that when Cu atoms in the core of Cu-Ag clusters, it will affect the strain distribution in clusters, also resulting in the different temperatures of packing transition [26]. Bochicchio et al. found that the melting temperature range of Cu@Ag nanoparticles with chiral structure is the same as that of highly symmetric pure clusters of the same size, indicating that the clusters have significant thermal stability [1]. In addition, some experiments have shown that the core-shell nanomaterials formed by Cu nanoparticles wrapped with Ag nanoparticles not only improve the oxidation resistance of Cu nanoparticles, but also maintain their excellent electrical properties including high electrical conductivity and high oxygen reduction reaction activity [27,28,29,30,31,32,33,34]. Because nanoalloy’s properties are significantly determined by their morphologies and atomic packing patterns in these particles, the knowledge about the structural phases at different temperatures during cooling becomes essential to use them in a variety of novel applications.

In this paper, molecular dynamics approaches based on the embedded atomic method (EAM) are used to simulate the changes of molten Cu_n_Ag_135−n_ (0–135) nanoclusters during the cooling process. Clusters containing 147 atoms usually present a perfect icosahedral geometry, and can be identified as typical “magic number clusters” in laboratory. When twelve atoms in this cluster are removed, the cluster containing 135 atoms may show a truncated icosahedral packing structure. Compared with the clusters containing 147 atoms, this cluster is close to spherical morphology in geometry, which is theoretic research significance. The individual clusters are analyzed based on the atomic average potential energy per atom, pair distribution functions, structural diagrams, visualization of the atomic packing structure, entropy, and free energy variation.

## 2. Model and Simulation

In this paper, the interaction among the atoms is described by the EAM form, which was proposed by Williams [35]. The total potential energy or internal energy of the system *E_tot_* is determined by

(1) $$E_{tot}=\frac{1}{2}\sum_{ij}V_{ij}\left(r_{ij}\right)+\sum_{i}F_{i}\left({\bar{\rho }}_{i}\right)$$

(2) $${\bar{\rho }}_{i}=\sum_{j\neq i}\rho_{j}\left(r_{ij}\right)$$

where $V_{ij}\left(r_{ij}\right)$ is the potential energy between atoms *i* and *j* having a distance of *r_ij_*, $F_{i}\left({\bar{\rho }}_{i}\right)$ is the embedded energy with an electron density of $\bar{\rho_{i}}$ at the position of the atom *i*. The density value is obtained from the superposition and sum of the electron density from the nearest neighboring atoms of the atom *i*. $\rho_{j}$ is the electron density of the neighbor atom j of the atom *i*.

The simulations were carried out in the NVT ensemble using Andersen thermostat. By solving Newton’s equations, we could obtain the positions and velocities of each atom, and a predictor-corrector algorithm was used to integrate equations of motion. Throughout the simulations, a time step of 1.6 × 10^−15^ s is used. At each temperature, the system was first fully equilibrated in 1.592 ns time before running to accumulate statistic, and the atomic trajectories and energy recorded in the subsequent 8 × 10^−^^3^ ns were used to obtain thermodynamic equilibrium values. The time steps used for statistics should be larger than 3N-6 in the simulated system, where N is the atom number in the system. Initially, we constructed a MD simulation cell with 20a_0_ × 20a_0_ × 20a_0_ (the Ag lattice constant a_0_ is 4.09 Å), and one cluster containing 135 atoms is put in the center of the cell. Here, the box size of the simulated central cell is large enough to avoid the interaction of the atoms in this central cell with the other atoms in its 26 neighbor imaging cells under periodic boundary conditions. Here, the distance between the central clusters in neighboring cells is 8.10 nm, which is much greater than the cutoff value of 0.60 nm for the interaction energy in the present binary system. The cluster containing 135 atoms was heated to a high temperature of 1250 K, and the cluster was melt after structural relaxation. Different molten nanoalloy clusters, as well as pure copper clusters were obtained by replacing Ag atoms in the pure silver clusters with 0–135 Cu atoms, respectively, and these clusters were subjected to structural relaxation at this high temperature. Subsequently, the different clusters obtained were subjected to a cooling process, that the temperature was lowered to 300 K at a decrement of 50 K. During cooling, the last step of the structure obtained from the structural relaxation at each temperature was simulated as the initial structure in the next temperature. In the present simulations, because of strong heat bath and very high surface/volume ratio for these clusters with a specific number, their configurations present high reproducibility during cooling. However, as the contained number of the atoms in clusters increases above 200, some of their structures show obvious variations in different simulations.

The following values were determined in the simulations.

(3) $$g\left(r\right)=\frac{1}{N^{2}}\langle \sum_{i\in N}\sum_{j\neq i\in N}\delta \left(r-r_{ij}\right)\rangle$$

where 〈·〉 denotes the average over the entire trajectory, and *N* is the atom number in this cell. *g*(*r*) is the pair distribution function, and gives the possibility of finding the atom pairs at a given distance *r*. When $r=r_{ij}$, *δ* is 1, whereas $r\neq r_{ij}$, it zero.

## 3. Results and Discussion

**Figure 1:**
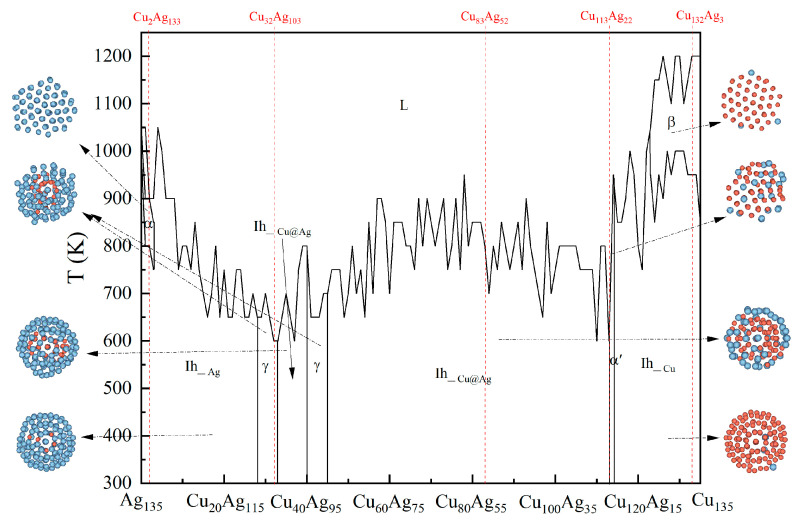
Structural diagram for Cu_n_Ag_135−n_ nanoalloy.

The diagram of these packing structures of Cu_n_Ag_135−n_ nanoalloys is shown in Figure 1, where the icosahedral structure is represented by Ih and the other packing structures by α, β, α’, γ, respectively. Additionally, silver atoms are marked in silvery grey and copper atoms in orange. As can be seen from this figure, the nanoparticles, having 1–3 Ag atoms replaced with Cu atoms, show a structural transition to α in the high temperature range, and then to icosahedral (Ih) geometry with the decrease in temperature. Here, α packing structure shows a “twins” structure having a feature of five-fold symmetry. As the number of replaced atoms increases to 4–27, the packing structure changes from disordered arrangement to icosahedral structure during the cooling process from 1200 K to 300 K, and the transition temperature shows a fluctuating decrease owing to the differences of atomic arrangement in the particle surface. Those clusters containing 34–40 Cu atoms, are also transformed to Cu@Ag packing patterns having an Ih configuration with decreasing the temperature. The clusters in the range of Cu_28_Ag_107_–Cu_33_Ag_102_ and Cu_41_Ag_94_–Cu_45_Ag_90_ are transformed from disordered to γ packing structures. Here, the Cu atoms in these clusters are arranged into incomplete icosahedral structure, while the Ag atoms are wrapped around those Cu atoms. It can be noted that their structural transition temperatures are lower than those of other clusters. The Cu@Ag nanoalloys having 46–112 Cu atoms are all changed from disordered packing to icosahedral structures. For the alloy cluster containing 113 Cu atoms, a α’ ordered structure occurs when the temperature is decreased to 600 K. In this structure, most of the Cu atoms are orderly arranged, while the Ag atoms and a small amount of Cu atoms are scattered in the outer layers of this cluster. The nanoclusters having 123–135 Cu atoms change into a β packing pattern at relative high temperatures, and then into stable Ih__Cu_ phase with decreasing the temperature further. Similar to the phase diagram of bulk Cu-Ag alloy, when the content of Ag or Cu in Cu_n_Ag_135−n_ clusters is large, the transition temperature is higher, while the transition temperature in the middle content region becomes lower. In addition, the transition temperature from liquid to crystal phase for pure Cu is apparently higher than that for Ag. Moreover, both Ag-rich and Cu-rich clusters have face centered cubic packing structure in high temperature range, but due to the small size, the clusters will present icosahedral structure with the decrease in temperature. There are also similar changes for Cu-Ag clusters containing 55 atoms, where the transition temperature of the clusters is also affected by the component content in the process of temperature change [26]. Qi et al. found that Ag_6_Cu_4_ forms a metallic glass while pure Cu cluster can form a FCC crystal for all quenching rates [36], suggesting that the component content plays a significant role in different cluster structures.

**Figure 2:**
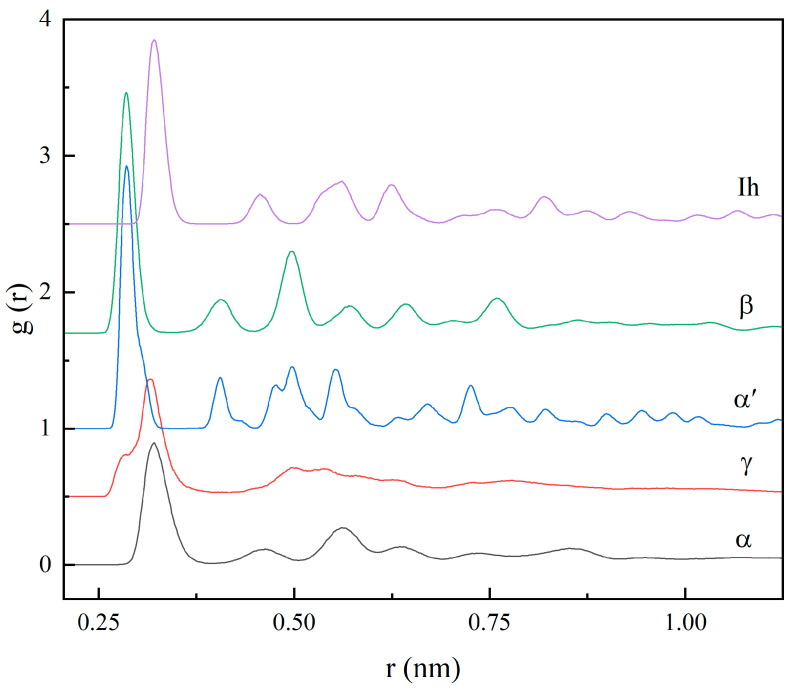
Pair-distribution functions (PDFs) of five packing structures.

Structural characteristics of these five packing patterns for α, γ, α’, β, and Ih as mentioned in the above diagram can be distinguished from PDF curves. As shown in Figure 2, for the α and β packing patterns, the PDFs present similar distinct peaks, though peak positions are different. These suggest that the atoms are packing in the similar structures, whereas the distances between atoms in the two patterns are different. For the γ packing structure, the first peak occurs at 0.32 nm, but the first main peak shows a splitting phenomenon. It indicates that the number of Cu-Cu atomic pairs increases with the increase in the number of Cu atoms, resulting in raising the number of short-range ordered local structures in these clusters. The other peaks on the curve are obviously connected with each other. These imply that there are more short-range ordered patterns in this packing structure, and multi-structures coexist in this cluster. The first peak of the PDF for the α’ packing structure is located at 0.29 nm. It is noteworthy that the shape of the first peak is not completely symmetric, which is due to the difference in the atomic distance between the atoms in the atom pairs close to the first nearest neighbor distance in the inner part of this cluster, where the distance between the atoms in the core of the cluster is slightly smaller than that in the outer region. The second and third peaks at 0.40 nm and 0.50 nm correspond to the positions of the second and third nearest neighbors. Splits appear on the two peaks, and many small peaks appear in the range of 0.63 nm to 1.01 nm. Compared with the other PDFs, there are clear and obvious peaks on the PDF for Ih packing structure. Here, the third peak at the position of 0.56 nm presents apparent difference from those PDFs for α and β. The reason is that the occupied volume of the Cu atom at the core of the cluster is smaller than that of the Ag atom. Meanwhile, the pairs’ distances formed by the Cu atom and other Ag atoms are mainly near the positions of the third nearest neighbor peak.

**Figure 3:**
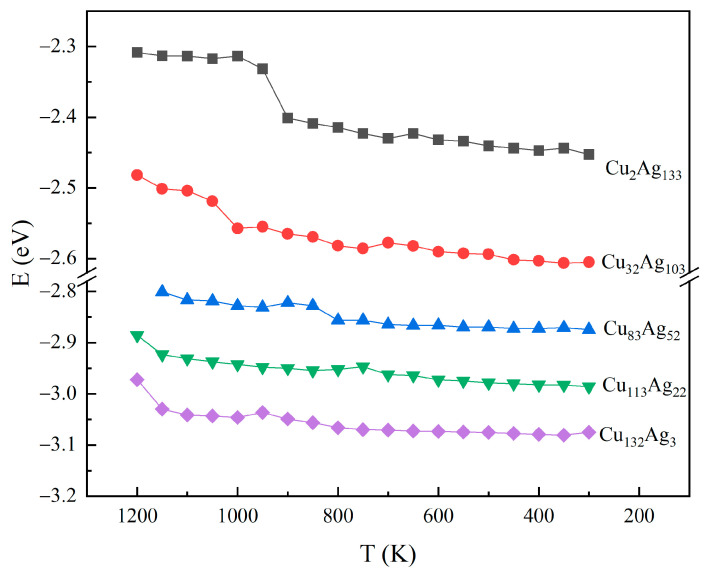
Variation of potential energy per atom of five clusters.

In order to further understand the structural transformation of these alloy clusters during the cooling process, five Cu@Ag nanoalloy clusters were selected, including Cu_2_Ag_133_, Cu_32_Ag_103_, Cu_83_Ag_52_, Cu_113_Ag_22_, and Cu_132_Ag_3_ clusters, which correspond to five packing patterns of α, γ, α’, β, and Ih, respectively. Figure 3 illustrates changes of potential energy per atom with the temperature during cooling. By comparing the average energy curves of the five clusters, it can be seen that the average energy of the nanoalloy clusters shows a decrease with the increase in the number of replaced Cu atoms in the Ag clusters. In addition, the energy of the five clusters all shows apparent decreases in some temperature ranges. For the Cu_2_Ag_133_ cluster, the energy decreases sharply between 950 K and 900 K, indicating that this cluster undergoes an apparently structural transformation. With the further decrease in temperature, the energy of the cluster almost remains unchanged. The energy curve of the Cu_32_Ag_103_ cluster suggests that there is an apparent structural transition between 1050 K and 1000 K, and the atoms adjust their positions until 650 K. Then, in the following temperatures below 600 K, the energy decreases slightly. For the Cu_83_Ag_52_ cluster, accompanied by a significant decrease at 800 K, disordered packing is changed to Ih structure, and the potential energy of this cluster remains essentially constant in the 300 K to 800 K range. The energy of the Cu_113_Ag_22_ cluster decreases slightly with decreasing temperature, from 1200 K to 1150 K, the shape of the cluster is elongated, and then a decreasing change occurs at 750 K–700 K. Correspondingly, some locally ordered arrangements appear in this cluster. Then, the cluster changes to a α’ packing structure in the 650 K–600 K. As the number of Cu atoms reaches 132, the energy decreases significantly in the temperature range of 1200 K to 1150 K, when the cluster change from disordered to β structure. When the temperature decreases to 900 K, an energy decrease occurs again.

**Figure 4:**
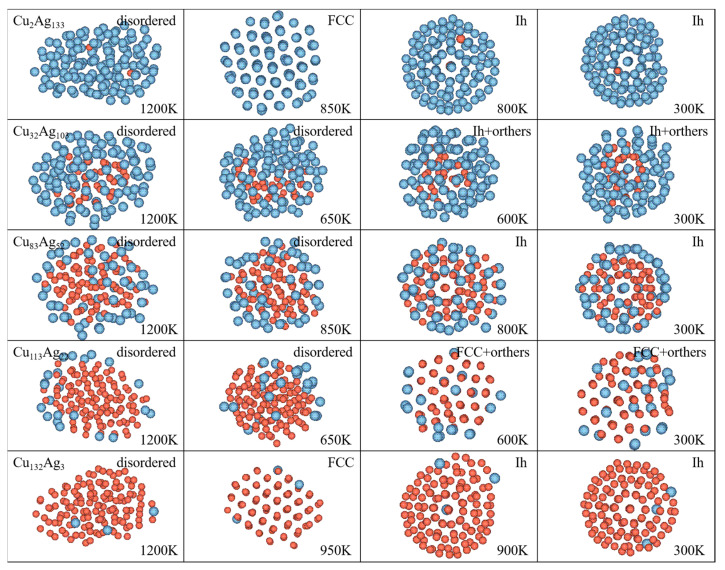
Packing structures of the clusters at different temperatures.

**Figure 5:**
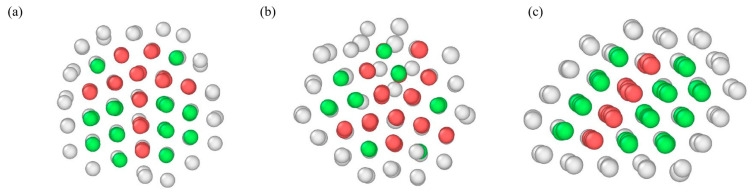
Structure type of the clusters at different temperatures. (**a**) Cu_2_Ag_133_ 850 K, (**b**) Cu_113_Ag_22_ 600 K, (**c**) Cu_132_Ag_3_ 950 K.

Figure 4 shows the packing structures of Cu_2_Ag_133_, Cu_32_Ag_103_, Cu_83_Ag_52_, Cu_113_Ag_22_, and Cu_132_Ag_3_ clusters at different temperatures. In the figure, silver atoms are marked in silvery grey and copper atoms in orange. For the Cu_2_Ag_133_ cluster at 850 K, it transforms from icosahedral structure to “five-fold twins” structure, where the atoms in different regions are face-centered cubic (FCC) packing, and they have commonly atomic arrangements in their interface. In Figure 5a, for the internal cross-section image of this cluster at this temperature, the atoms in face-centered cubic (FCC) packing is represented by green, the interfacial atoms in hexagonal close-packed structure (HCP) red, and the other structures gray for these atoms in the surface. When the temperature decreases to 800 K, this cluster changes from the α packing structure to a Ih structure. At 300 K, the cluster still remains the Ih structure. It can be noted that although this structure is more compact compared to that at 800 K, the outer shell of the cluster shows defects. The Cu_32_Ag_103_ cluster is disordered at 1200 K, and there are still many local structures in different small parts at 650 K. At 600 K, the Cu atoms in the cluster’s inner part are arranged into incomplete icosahedral structure, while the Ag atoms are wrapped around the Cu atoms to form outer shells. When the temperature drops to 300 K, the Cu atoms in the center of this cluster are arranged more compactly. As for the Cu_83_Ag_52_ cluster, the overall cluster shows contraction at 1200 K down to 850 K, and transforms to Ih structure at 800 K, where the Ag atoms are loosely arranged in the outer shell. At 300 K, the cluster is still the Ih structure, and some Cu and Ag atoms interchange their positions. The Cu_113_Ag_22_ cluster transforms into the α’ packing structure at 600 K. From Figure 5b, it can be seen that the α’ packing structure is mainly composed of FCC and HCP structures, but they are not symmetrically arranged, which is somewhat different from the α packing structure. Cu_132_Ag_3_ nanoalloy has a layered arrangement stacking structure at 950 K, most of the atoms of the β packing structure are in FCC structure and a few atoms are in HCP structure as seen from Figure 5c. When the temperature decreases to 900 K, the clusters change from β packing structure to the Ih packing structure, and this cluster keeps the Ih structure until room temperature.

**Figure 6:**
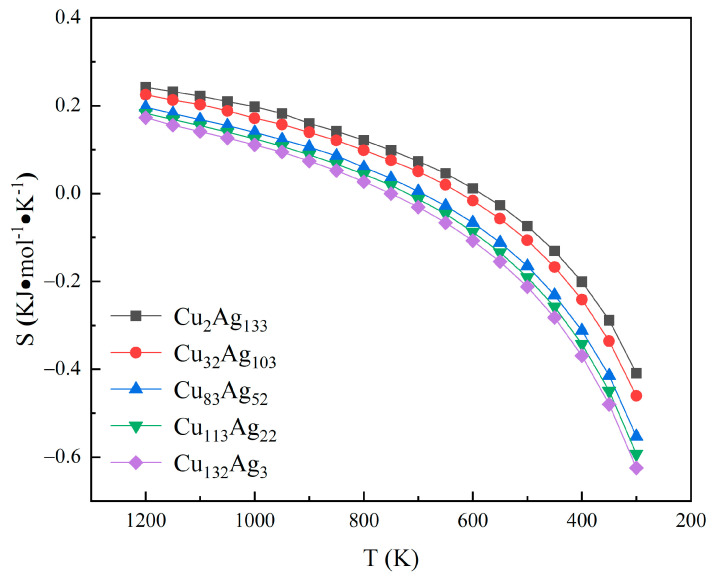
Entropy curves of five clusters with temperature.

The entropy as a function of temperature can be obtained by subtracting the free energy from the potential energy and dividing the difference by the temperature, as shown in Figure 6. It can be used as a measure of the degree of order, i.e., for a given system, the greater the degree of disorder in the atomic arrangement, the greater the entropy value [37]. As can be seen from the figure, the entropy values of the five clusters firstly decrease slowly with the decrease in the temperature, and then decrease rapidly, and the entropy values reach negative values, indicating that these clusters are arranged in an orderly manner. Among the five clusters, the entropy of the Cu_2_Ag_133_ cluster is significantly higher than the other clusters, followed by Cu_32_Ag_103_, Cu_83_Ag_52_, Cu_113_Ag_22_, and the lowest of the Cu_132_Ag_3_ cluster. These suggest that the number of replaced Cu atoms in the Ag clusters has an effect on the overall entropy value of the clusters.

**Figure 7:**
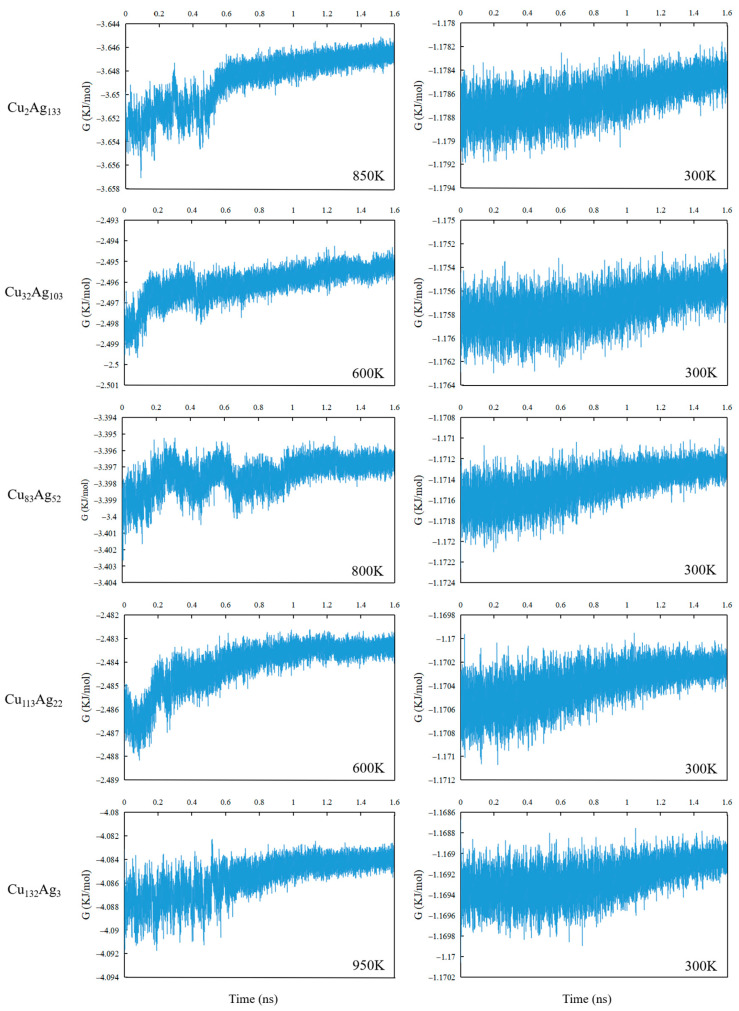
Free energy variation of five clusters with time.

Figure 7 shows the rotational free energy variation of the five clusters Cu_2_Ag_133_, Cu_32_Ag_103_, Cu_83_Ag_52_, Cu_113_Ag_22_, and Cu_132_Ag_2_ at different temperatures with time. The free energy referred to here is composed of translational free energy and rotational free energy. At one temperature, the rotational free energy varies significantly, while the translational motion is temperature dependent, and its value is a constant. The free energy from the rotation is given by −*k_B_*T × ln*Zr*, where *k_B_* is the Boltzmann constant, T temperature, and *Zr* rotational partition function. For the Cu_2_Ag_133_ cluster, the free energy of the cluster fluctuates in the 0 to 0.5 ns time range at 850 K, increases significantly in the subsequent 0.1 ns, and then gradually flattens out. At 600 K, the free energy of Cu_32_Ag_103_ cluster fluctuates more significantly in the 0 to 0.1 ns and 0.45 to 0.5 ns time ranges, except for the pronounced fluctuations in the other time, and tends to level off in the other time. In these time regimes that the free energy values change more significantly, the conformations of the cluster change significantly, indicating the occurrences of packing transition. The free energy of Cu_83_Ag_52_ clusters at 800 K showed a largely fluctuating between 0 and 0.95 ns, suggesting that followed conformational variations in this time regime, atomic packing becomes stable. At 600 K, the free energy of Cu_113_Ag_22_ clusters show that the packing transition occurs in a short time regime of 0.35 nm. For the Cu_132_Ag_3_ cluster, at 950 K, the free energy changes dramatically at the time range of 0–0.6 ns, suggesting occurrences of a large number of conformations. The free energy changes of the five clusters at 300 K are similar, i.e., they increase slowly with increasing time, where the Cu_2_Ag_133_ cluster has the smallest free energy value, followed by the Cu_32_Ag_103_, Cu_83_Ag_52_, Cu_113_Ag_22_ clusters, and the Cu_132_Ag_2_ cluster has the largest free energy value.

## 4. Conclusions

We performed molecular dynamics calculations for the packing structures and thermodynamics of Cu_n_Ag_135−n_ (n = 0–135) nanoalloys during cooling. With the increase in Cu-content in the alloy cluster, as well as decreasing the temperature, the binary nanoalloy shows a variety of packing structures. When the number of Ag atoms in the cluster is relatively high, the Cu@Ag nanoclusters basically exhibit Ih and γ two packing structures in the low temperature region. Although the number of Cu atoms accounts for more than 33%, the nanoalloys exhibit icosahedral structure in the low temperature region. When there is only a few of Ag atoms or Cu atoms in Cu@Ag nanoclusters, the clusters show α or β packing structures in the high temperature region, and then transform into Ih packing. For these nanoclusters, when the number of Cu atoms is less than 112, the Cu@Ag nanoclusters can be formed. The changes of entropy for the Cu-Ag nanoalloys are influenced by the number of replaced Cu atoms, and indicate the order degree in these alloys. As the number of replaced Cu atoms in Ag clusters increases, the entropy and free energy of the alloy clusters increase. Accompanying with the transition of packing structure at different temperatures, the changes of rotation free energy can also be used to indicate the transition.

## Data Availability

Not applicable.

## Sample Availability

Samples of the compounds are available from the authors.

## References

[1] Bochicchio D., Ferrando R. (2012). Structure and thermal stability of AgCu chiral nanoparticles. Eur. Phys. J. D.

[2] Langlois C., Li Z.L., Yuan J. (2012). Transition from core–shell to Janus chemical configuration for bimetallic nanoparticles. Nanoscale.

[3] Baletto F., Mottet C., Ferrando R. (2003). Growth of three-shell onionlike bimetallic nanoparticles. Phys. Rev. Lett..

[4] Shin K., Da H.K., Sang C.Y. (2012). Structural stability of AgCu bimetallic nanoparticles and their application as a catalyst: A DFT study. Catal. Today.

[5] Yun H.J., Jung I., Na R.K. (2012). Synthesis and characterization of highly conductive Sn–Ag bimetallic nanoparticles for printed electronics. J. Nanopart. Res..

[6] Sobrinho D.G., Nomiyama R.K., Chaves A.S. (2015). Structure, electronic, and magnetic properties of binary Pt_n_TM_55-n_ (TM = Fe, Co, Ni, Cu, Zn) nanoclusters: A density functional theory investigation. J. Phys. Chem. C.

[7] Ferrando R. (2016). Structure and Properties of Nanoalloys Volume 10||Optical Properties of Nanoalloys.

[8] Li G., Qiang W., Li D. (2009). Size and composition effects on the melting of bimetallic Cu-Ni clusters studied via molecular dynamics simulation. Mater. Chem. Phys..

[9] Rossi G., Rapallo A., Mottet C. (2004). Magic polyicosahedral core-shell clusters. Phys. Rev. Lett..

[10] Ferrando R., Jellinek J., Johnston R.L. (2008). Nanoalloys: From theory to applications of alloy clusters and nanoparticles. Chem. Rev..

[11] Rapallo A., Rossi G., Ferrando R., Fortunelli A., Curley B.C., Lloyd L.D. (2005). Global optimization of bimetallic cluster structures: I. Size-mismatched Ag-Cu, Ag-Ni, and Au-Cu systems. J. Chem. Phys..

[12] Yang J., Hu W., Wu Y. (2012). Substrate dependence of growth configurations for Co-Cu bimetallic clusters. Cryst. Growth Des..

[13] Chandross M. (2014). Energetics of the formation of Cu-Ag core-shell nanoparticles. Model. Simul. Mater. Sci. Eng..

[14] Rui W. (2010). The study and application of core-shell structure bimetallic nanoparticles. Prog. Chem..

[15] Bochicchio D., Ferrando R. (2013). Morphological instability of core-shell metallic nanoparticles. Phys. Rev. B.

[16] Fakhri A., Tahami S., Naji M. (2017). Synthesis and characterization of core-shell bimetallic nanoparticles for synergistic antimicrobial effect studies in combination with doxycycline on burn specific pathogens. J. Photochem. Photobiol. B.

[17] Fedrigo S., Harbich W., Belyaev J. (1993). Evidence for electronic shell structure of small silver clusters in the optical absorption spectra. Chem. Phys. Lett..

[18] Terry L.R., Pudney C.R., Gersen H. (2018). The effect of isotropic pressure on the electronic structure and superatomic orbitals of molecular [Ag_44_(SPhCOOH)_30_]^4−^, [Ag_44_(SPhF_2_)_30_]^4−^ & [Ag_25_(SPhMe_2_)_18_]^−^ Nanoclusters. arXiv.

[19] Jalili S., Goliaei E.M., Schofield J. (2017). Silver cluster supported on nitrogen-doped graphene as an electrocatalyst with high activity and stability for oxygen reduction reaction. Int. J. Hydrogen Energy.

[20] Buratto S.K. (2007). Au_n_ and Ag_n_ (n = 1–8) nanocluster catalysts: Gas-phase reactivity to deposited structures. Chem. Phys. Solid Surf..

[21] Chaiendoo K., Tuntulani T., Ngeontae W. (2015). A highly selective colorimetric sensor for ferrous ion based on polymethylacrylic acid-templated silver nanoclusters. Sens. Actuators B Chem..

[22] Wang Y., Dai C., Yan X.P. (2014). Fabrication of folate bioconjugated near-infrared fluorescent silver nanoclusters for targeted in vitro and in vivo bioimaging. Chem. Commun..

[23] Wen T., Qu F., Li N.B. (2012). Polyethyleneimine-capped silver nanoclusters as a fluorescence probe for sensitive detection of hydrogen peroxide and glucose. Anal. Chim. Acta.

[24] Liang M., Xiong Z. (2020). Surface evolution of CuAg bimetallic systems: From experiments to molecular dynamics simulation. J. Phys. Chem. C.

[25] Sarkar J., Bhattacharyya M., Kumar R. (2016). Synthesis and characterizations of Cu-Ag core-shell nanoparticles. Adv. Sci. Lett..

[26] Liu J.H., Zhang L. (2020). Strain-induced packing transition of Ih Cu_n_@Ag_55−n_(n = 0, 1, 13, 43) clusters from atomic simulations. Math. Biosci. Eng..

[27] Gao B., Gao J., Jiang H. (2000). Plating structure and antioxygenation of micron Cu-Ag bimetallic powder. Acta Phys. Chem. Sin..

[28] Tsai C.H., Chen S.Y., Song J.M. (2013). Thermal stability of Cu@Ag core–shell nanoparticles. Corros. Sci..

[29] Lee C., Kim N.R., Koo J. (2015). Cu-Ag core–shell nanoparticles with enhanced oxidation stability for printed electronics. Nanotechnology.

[30] Grouchko M., Kamyshny A., Magdassi S. (2009). Formation of air-stable copper-silver core-shell nanoparticles for inkjet printing. J. Mater. Chem. A.

[31] Li W.L., Hu D.W. (2017). Printable and flexible copper-silver alloy electrodes with high conductivity and ultrahigh oxidation resistance. Appl. Mater. Interfaces.

[32] Kim S.J., Stach E.A., Handwerker C.A. (2010). Fabrication of conductive interconnects by Ag migration in Cu-Ag core-shell nanoparticles. Appl. Phys. Lett..

[33] Zhang N., Chen F., Wu X. (2017). Activity origin of core-shell and alloy AgCu bimetallic nanoparticles for oxygen reduction reaction. J. Mater. Chem. A.

[34] Wu X., Chen F., Zhang N. (2017). Activity trends of binary silver alloy nanocatalysts for oxygen reduction reaction in alkaline media. Small.

[35] Williams P.L., Mishin Y., Hamilton J.C. (2006). An embedded-atom potential for the Cu-Ag system. Model. Simul. Mater. Sci. Eng..

[36] Qi Y., Çağın T., Kimura Y., Goddard W.A. (1999). Molecular-dynamics simulations of glass formation and crystallization in binary liquid metals: Cu-Ag and Cu-Ni. Phys. Rev. B.

[37] Jiang Y.Q. (2020). Simulation and analysis of melting behavior of local atomic structure of refractory metals vanadium. Acta Phys. Sin..

